# Dihydro-CDDO-Trifluoroethyl Amide (dh404), a Novel Nrf2 Activator, Suppresses Oxidative Stress in Cardiomyocytes

**DOI:** 10.1371/journal.pone.0008391

**Published:** 2009-12-21

**Authors:** Tomonaga Ichikawa, Jinqing Li, Colin J. Meyer, Joseph S. Janicki, Mark Hannink, Taixing Cui

**Affiliations:** 1 Department of Cell Biology and Anatomy, University of South Carolina School of Medicine, Columbia, South Carolina, United States of America; 2 Department of Pharmacology, Reata Pharmaceuticals, Inc., Irving, Texas, United States of America; 3 Department of Biochemistry, University of Missouri - Columbia, Columbia, Missouri, United States of America; University of Illinois at Chicago, United States of America

## Abstract

Targeting Nrf2 signaling appears to be an attractive approach for the treatment of maladaptive cardiac remodeling and dysfunction; however, pharmacological modulation of the Nrf2 pathway in the cardiovascular system remains to be established. Herein, we report that a novel synthetic triterpenoid derivative, dihydro-CDDO-trifluoroethyl amide (dh404), activates Nrf2 and suppresses oxidative stress in cardiomyocytes. Dh404 interrupted the Keap1-Cul3-Rbx1 E3 ligase complex-mediated Nrf2 ubiquitination and subsequent degradation saturating the binding capacity of Keap1 to Nrf2, thereby rendering more Nrf2 to be translocated into the nuclei to activate Nrf2-driven gene transcription. A mutant Keap1 protein containing a single cysteine-to-serine substitution at residue 151 within the BTB domain of Keap1 was resistant to dh404-induced stabilization of Nrf2 protein. In addition, dh404 did not dissociate the interaction of Nrf2 with the Keap1-Cul3-Rbx1 E3 ligase complex. Thus, it is likely that dh404 inhibits the ability of Keap1-Cul3-Rbx1 E3 ligase complex to target Nrf2 for ubiquitination and degradation via modifying Cys-151 of Keap1 to change the conformation of the complex. Moreover, dh404 was able to stabilize Nrf2 protein, to enhance Nrf2 nuclear translocation, to activate Nrf2-driven transcription, and to suppress angiotensin II (Ang II)-induced oxidative stress in cardiomyocytes. Knockdown of Nrf2 almost blocked the anti-oxidative effect of dh404. Dh404 activated Nrf2 signaling in the heart. Taken together, dh404 appears to be a novel Nrf2 activator with a therapeutic potential for cardiac diseases via suppressing oxidative stress.

## Introduction

Triterpenoids, synthesized in many plants, including a large variety of vegetarian foods and medicinal herbs, are widely used as traditional medicine of in Asian countries [1]. More than 20,000 triterpenoids exist in nature [2]. In fact, triterpenoids of oleanolic acid (OA) and ursolic acid (UA) have emerged to be attractive drug scaffolds because they are relatively non-toxic, cytoprotective, hypoglycemic, anti-inflammatory, anti-hyperlipidemic, and anti-tumorigenic [1]. To improve the pharmacological potency, novel derivatives of OA, such as 2-cyano-3,12-dioxooleana-1,9(11)-dien-28-oic acid (CDDO), CDDO methyl ester (CDDO-Me), CDDO imidazolides (CDDO-Im), and CDDO amides (methyl amide, CDDO-MA; ethyl amide, CDDO-EA), have been synthesized [3]. These synthetic derivatives of OA are potent multifunctional molecules *in vitro*. Depending on the dose, they can suppress inflammation, activate cytoprotective pathways, induce differentiation, inhibit proliferation and induce apoptosis. In fact, they are the most potent anti-inflammatory and anti-carcinogenic triterpenoinds identified [4]. Importantly, CDDO-Me that has been tested in clinical oncology trails is currently in a phase IIb clinical trial for the treatment of chronic kidney diseases. However, the therapeutic potential of synthetic oleanane triterpenoids (SO) in cardiovascular diseases has not been explored.

Molecular mechanisms underlying the pleiotropic nature of SO are only partly understood. However; it has been recently demonstrated that activation of nuclear factor-erythroid (NF-E) 2–related factor 2 (Nrf2) signaling is critical for the SO-mediated anti-inflammatory and anti-carcinogenic activities [4].

Nrf2 is a member of the Cap ‘n’ Collar (CNC) family of basic leucine zipper (bZip) transcription factors that includes NF-E2, Nrf1-3 and Bach1-2 [5]–[9]. As other members of the CNC family of bZip transcription factors, Nrf2 forms a heterodimer with its obligatory partner Maf, thereby binding to a *cis*-acting enhancer sequence known as the antioxidant response element (ARE), also referred to as the electrophile response element (EpRE) with a core nucleotide sequence of 5′-RTGACNNNGC-3′, to regulate the basal and inducible expression of more than 200 genes that can be grouped into several categories including antioxidant genes, phase II detoxifying enzymes, transporters, scavenger receptor, chaperone proteins, transcription factors [10]–[12]. The protein stability and transcriptional activity of Nrf2 is principally regulated by a BTB-Kelch protein, Keap1 that functions as a substrate adaptor for a cullin (Cul)3-dependent E3 ubiquitin ligase complex [10]–[12]. Keap1 targets Nrf2 for ubiquitination and subsequent degradation by the 26S proteasome. Mounting evidence has revealed that Nrf2 is a major regulator of cellular defenses against various pathological stresses in different organs including lung, liver, gastrointestinal tract, bladder, kidney, brain, skin, and ovary. Nrf2 also appears to be an attractive drug target for the treatment or prevention of several human disorders such as pulmonary fibrosis, hepatic and gastrointestinal disease, carcinogenesis and neurodegenerative disease [12]–[23]. Of note, we have recently demonstrated that Nrf2 is a critical negative regulator of pathological cardiac hypertrophy and heart failure via its ability to orchestrate a group of antioxidant genes [24]. These results suggest that targeting Nrf2 signaling might be a novel therapeutic strategy for the treatment of maladaptive cardiac hypertrophy and the prevention of heart failure. Interestingly, a recent report highlighted the fact that the Keap1-Nrf2 system comprises discrete sensor sites, including the Keap1 cysteines Cys-151 and Cys-275, for a variety of Nrf2-activating compounds and special “cysteine codes” are linked with the Nrf2-mediated biological actions [25]. Therefore, a novel class of synthetic SO might activate the Nrf2-driven cardiac protective signaling via modulating “cysteine codes” of Keap1-Nrf2 system.

In the present study, we report that a novel triterpenoid derivative of dihydro-CDDO-trifluoroethyl amide (dh404) stabilizes Nrf2 protein via modulating Keap1 Cys-151, thereby activating Nrf2-driven transcriptional activity. In addition, dh404 inhibits the hypertrophic agonist angiotensin II (ANG II)-induced formation of reactive oxygen species (ROS) and reactive nitrogen species (RNS) in cardiomyocytes via the activation of Nrf2 signaling. Notably, dh404 activates Nrf2 signaling in the heart. Our results highlight a therapeutic potential of dh404 for the treatment of heart disease.

## Results

### Dh404 Activates Nrf2 by Disrupting Keap1-Dependent Suppression of Nrf2

**Figure 1:**
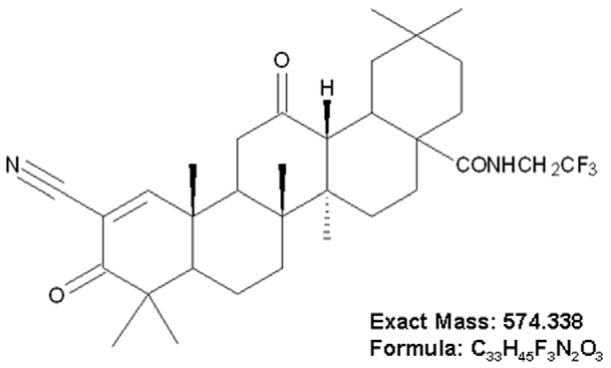
Structure of dh404. A novel dihydro-CDDO-trifluoroethyl amide (dh404) of 2-cyano-3,12-dioxooleana-1-ene-28-trifluoroethylamide (C_33_H_45_F_3_N_2_O_3_) with a molecular weight of 574.338.

**Figure 2:**
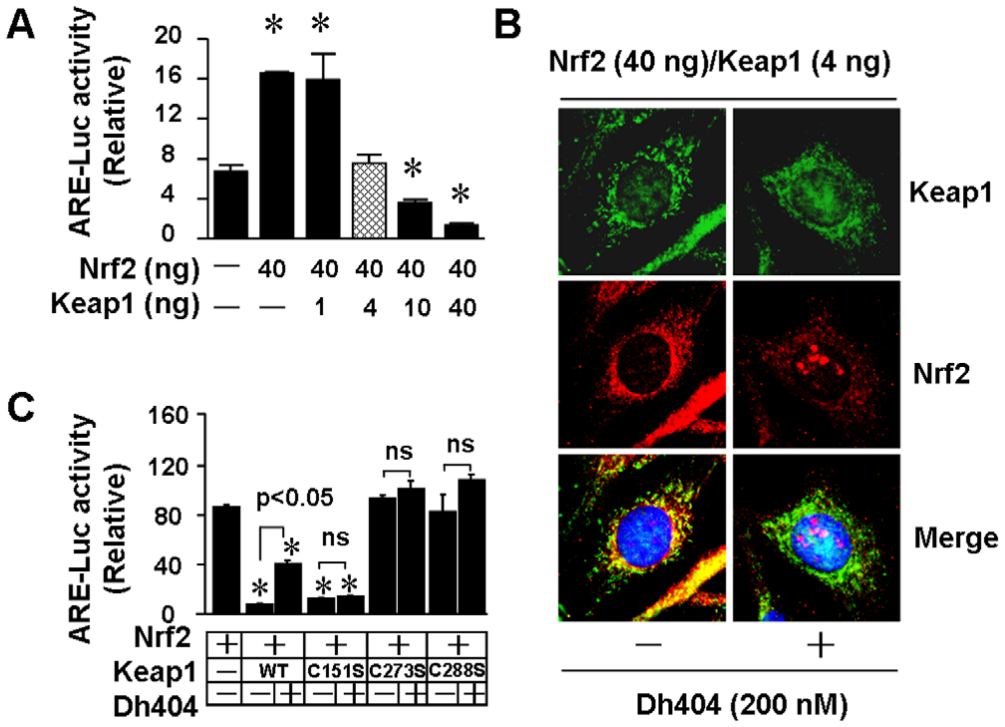
Regulation of Keap1-Nrf2 Homeostasis by dh404. *A*, Titration of Keap1-mediated suppression of Nrf2 transcritional activity. NIH 3T3 cells were transfected with plasmids containing an ARE-dependent firefly luciferase reporter gene (ARE-Luc), expression plasmids for wild-type HA-Nrf2 and CBD-Keap1, and plasmids encoding *Renilla* luciferase to normalize transfection efficiency. The transfections were performed with 100 ng of ARE-Luc plasmid, 10 ng of pRL-TK, 40 ng of HA-Nrf2, and 1–40 ng of CBD-Keap1, as well as different amounts of pcDNA3.1 to maintain a total level of plasmid DNA at 200 ng. ARE reporter activity was measured 24 hours after the transfection. Results are representative of three independent experiments. *p<0.05 vs control pcDNA3.1. *B*, Effect of dh404 on Nrf2 nuclear translocation. NIH 3T3 cells were transfected for 24 hours with plasmids of HA-Nrf2 and CDB-Keap1 with a ratio of 10∶1 that resulted in complete suppression of Nrf2 transcriptional activity as indicated in *A*. The transfected cells were treated with dh404 (200 nM) in serum-free DMEM for 2 hours, and then subjected to immunofluorescence cofocal microscopic analysis. *C*, Cysteine residues in Keap1 required for dh404-mediated Nrf2 activation. NIH 3T3 cells were transfected for 24 hours with plasmids of HA-Nrf2 and CDB-Keap1 or plasmids of mutant Keap1 containing single cysteine-to-serine substitutions with a ratio of 10∶1 as indicated. The transfected cells were treated with or without dh404 (200 nM) in serum-free DMEM for 6 hours prior to ARE reporter assay. Results are representative of three independent experiments. *p<0.05 vs Nrf2 transfected group.

It has been well documented that synthetic triterpenoid derivatives are able to activate Nrf2 [4], therefore, we synthesized a novel triterpenoid derivative of dihydro-CDDO-trifluoroethyl amide that was named dh404 (Figure 1) and investigated the impact of dh404 on Keap1-Nrf2 homeostasis. Firstly, we established a system of Keap1-Nrf2 homeostasis in NIH 3T3 cells that express negligible levels of Nrf2 and Keap1 as previously described [26]. Briefly, different ratios of Keap1 and Nrf2 plasmids were transfected into NIH 3T3 cells. The Nrf2-driven transcriptional activity reached the basal level when the exogenous expression of Nrf2 and Keap1 was achieved in a ratio of 10∶1 (Nrf2:Keap1) (Figure 2A). Confocal microscopic analysis clearly indicated that HA-Nrf2 protein was efficiently sequestered in the cytoplasm by the co-expression of CBD-Keap1 protein (Figure 2B). Thus, we used the balanced Keap1-Nrf2 expression system in the present study. Secondly, we determined the potential of dh404 to activate Nrf2. As shown in Figure 2B, dh404 triggered Nrf2 nuclear translocation, suggesting that dh404 is able to activate Nrf2. We have previously demonstrated that C273 and C288 which are distinct cysteine residues in Keap1, are required for Keap1-mediated constitutive suppression of Nrf2 activity, and C151, that is required for escape by Nrf2 from Keap1-dependent suppression in response to sulforaphane and oxidative stress [26]. Accordingly, the effect of dh404 on Nrf2 transcriptional activity was determined in the cells transfected with Nrf2, Keap1, and these Keap1 mutants (Figure 2C). Dh404 activated Nrf2 transcriptional activity in the cells transfected with Nrf2 and Keap1 but not with Nrf2 and C151S Keap1 mutant, suggesting that dh404 specifically interacts with the Cys-151 residue in Keap1 leading to disruption of the Keap1-dependent suppression of Nrf2.

### Dh404 Inhibits Keap1-Mediated Nrf2 Degradation by Interfering with the Ability of Keap1-Cul3-Rbx1 E3 Ligase Complex to Target Nrf2 for Ubiquitination

To explore the molecular mechanism by which dh404 disrupts the Keap1-mediated suppression of Nrf2, we examined effect of dh404 on the Keap1-mediated Nrf2 ubiquitination and subsequent proteosome-mediated degradation as well as the assembling of Keap1-Cul3-Rbx1 E3 ligase complex for the Nrf2 ubiquitination and degradation in HEK293 cells as previously described [27].

**Figure 3:**
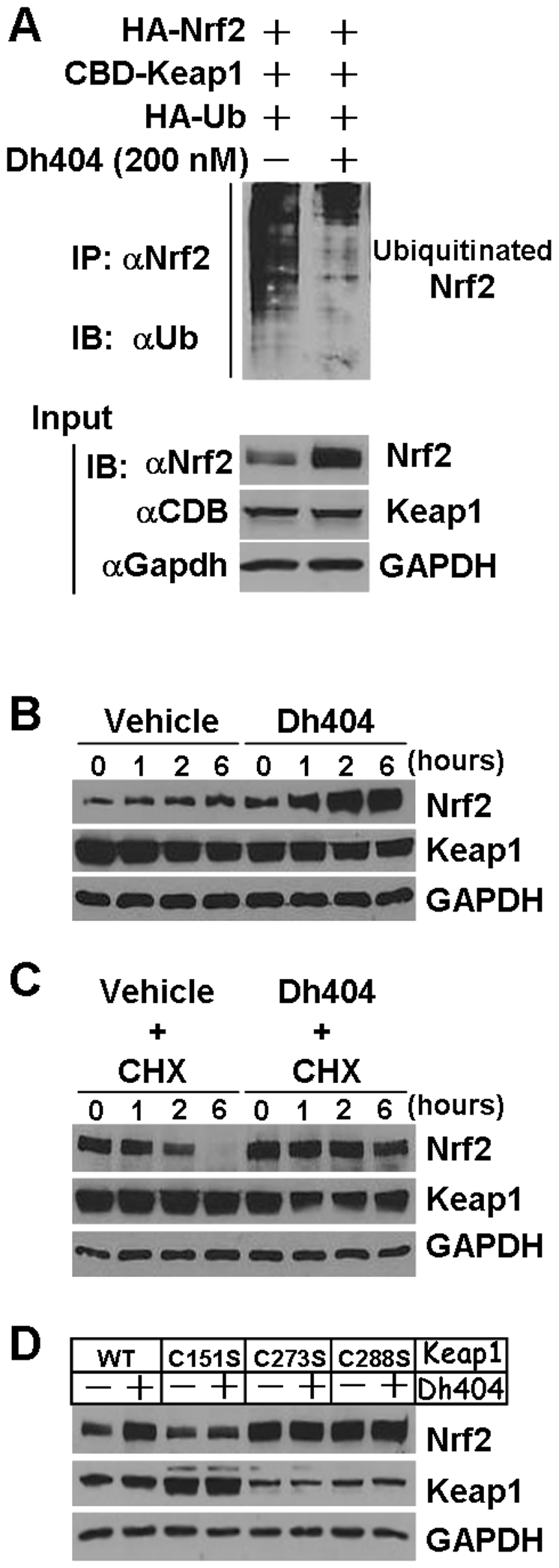
Effect of dh404 on Keap1-mediated Nrf2 ubiquitination and degradation. *A*, HEK 293 cells that were transfected with plasmids of HA-Nrf2 (1000 ng), CBD-Keap1 (100 ng) and HA-Ub (1000 ng) for 24 hours were treated with or without dh404 (200 nM) in DMEM with 10% FBS for 2 hours as indicated. Ubiquitination of Nrf2 was determined by immunoprecipitation (IP) and immunoblot (IB) analysis using appropriate antibodies as indicated. Inputs of 10 µg of whole cell lysates were utilized to monitor the protein expression levels of target genes. *B* & *C*, HEK 293 cells that were transfected with plasmids of HA-Nrf2 (800 ng) and CBD-Keap1 (80 ng) for 24 hours were treated with or without dh404 (200 nM) and/or CHX (10 µg/ml) in DMEM with 10% FBS as indicated. D, HEK293 Cells were transfected with HA-Nrf2, CBD-Keap1 or mutant Keap1 as described above, and then stimulated with or without dh404 (200 nM) for 2 hours. Whole cell lysates were subjected to immunoblot analysis using anti(α)Nrf2, αKeap1 or αGapdah, as indicated. Results are representative of three independent experiments.

We have demonstrated that Nrf2 protein turnover in cells is very fast and most of Nrf2 proteins are rapidly degraded via the ubiquitin-proteosome system [27]. Of interest, we observed that dh404 treatment resulted in a decrease in the Nrf2 ubiquination (Figure 3A). Moreover, dh404 suppressed Keap1-mediated Nrf2 degradation via a posttranscriptional modulation at the C151 residue in Keap1 (Figures 3B–D). These results indicate that dh404 interfered with Keap1-dependent machinery for Nrf2 ubiquination and degradation via its ability to modulate Cys-151 in Keap1.

**Figure 4:**
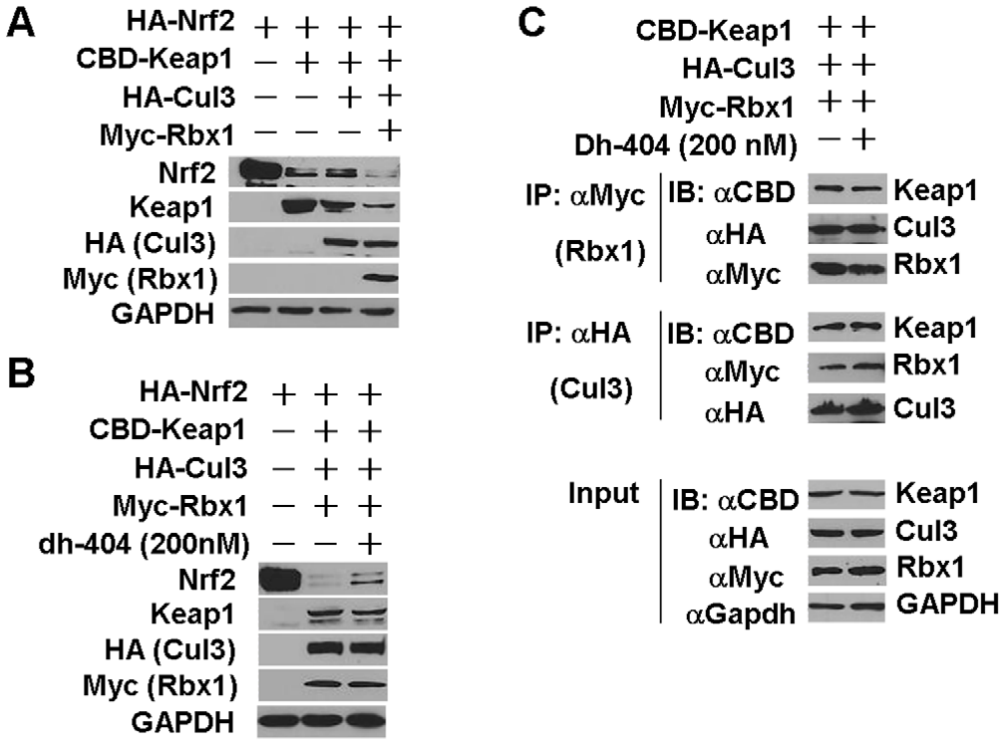
Effect of dh404 on the Keap1/Cul3/Rbx1 complex-mediated Nrf2 degradation and on the assembling of Keap1/Cul3/Rex1 complex. HEK293 cells were transfected with plasmids of HA-Nrf2 (1000 ng), CBD-Keap1 (100 ng), HA-Cul3 (500 ng) or Myc-Rbx1 (400 ng) for 24 hours as indicated, and then were treated with or without dh404 (200 nM) in DMEM with 10% FBS for 2 hours as indicated.*A*, Recapitulation of Keap1/Cul3/Rbx1 complex-mediated Nrf2 degradation in HEK293 cells. *B*, Effect of dh404 on Keap1/Cul3/Rbx1 complex-mediated Nrf2 degradation in HEK293 cells. *C*, Effect of dh404 on assembling of Keap1/Cul3/Rbx1 complex in HEK293 cells. Cell lysates were subjected to immunoprecipitation (IP) and IB analysis using antibodies of Nrf2, Keap1, HA, Myc, and Gapdh as indicated. Results[Sec s2] are representative of three independent experiments.

We and others have demonstrated that Keap1 functions as a substrate adaptor protein for a Cul3/Rbx1 E3 ligase complex to target Nrf2 ubiquitination and subsequent proteosome-mediated degradation [27]–[29]. Therefore, the effect of dh404 on the Keap1-Cul3-Rbx1complex-mediated Nrf2 degradation and the complex assembling were determined. The input amounts of plasmids utilized for transfections including CBD-Keap1, HA-Cul3 andMyc-Rbx1 were carefully titrated, so that the steady-state levels of Nrf2 protein expression were close to basal levels (Figure 4A). Under this condition, dh404 treatment led to a dramatic increase in Nrf2 protein levels, suggesting that dh404 is able to inhibit the Keap1-Cul3-Rbx1 dependent Nrf2 degradation (Figure 4B). Moreover, reciprocal immunoprecipitation and immunobot analysis demonstrated that dh404 did not disrupt a complex formation of Keap1-Cul3-Rbx1 in HEK293 cells (Figure 4C).

### Dh404 Increases Nrf2 Nuclear Translocation Independent of Keap1

**Figure 5:**
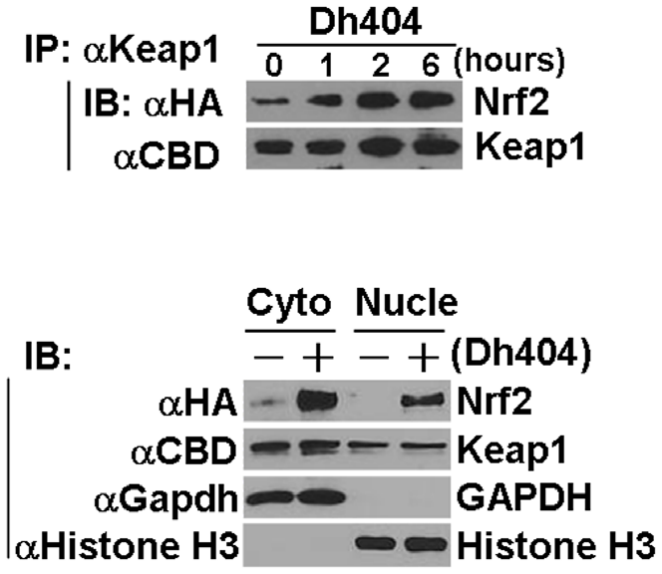
Regulation of interaction of Nrf2 with Keap1 by dh404. HEK 293 cells were transfected with HA-Nrf2 and CBD-Keap1 as described in Figure 3B, and treated with dh404 (200 nM) in DMEM with 10% FBS as indicated. Immunoprecipitation (IP) and immunoblot (IB) analysis were performed using appropriate antibodies as indicated. Results are representative of three separated experiments. Cyo, cytosolic proteins; Nucle, nuclear proteins.

Several lines of evidence have revealed that a relative slow turnover of Nrf2 in the nucleus involves Keap1-dependent and –independent mechanisms [30]–[35]. Accordingly, we examined whether or not dh404 regulates Nrf2-Keap1 nuclear shuttling. While dh404 enhanced the association of Nrf2 and Keap1 and facilitated Nrf2 nuclear accumulation, it did not increase the nuclear levels of Keap1 (Figure 5). These results suggest that dh404-induced Nrf2 nuclear translocation is via a mechanism independent of Keap1.

Collectively, we have demonstrated that dh404 interrupts the Keap1-Cul3-Rbx1 E3 ligase complex-mediated Nrf2 ubiquitination and subsequent degradation thereby saturating the binding capacity of Keap1 to Nrf2 and rendering more Nrf2 to be translocated into nuclei to activate Nrf2-driven gene transcription. Since dh404 does not dissociate the interaction of Nrf2 with the Keap1-Cul3-Rbx1 E3 ligase complex, it is likely that dh404 inhibits the ability of Keap1-Cul3-Rbx1 E3 ligase complex to target Nrf2 for ubiquitination and degradation via changing the conformation of the complex. Moreover, Cys151 of Keap1 is the primary sensor for dh404-mediated activation of Nrf2 signaling.

### Dh404 Stabilizes Nrf2 and Activates Nrf2-Driven Transcriptional Activity in H9C2 Cardiomyocytes

**Figure 6:**
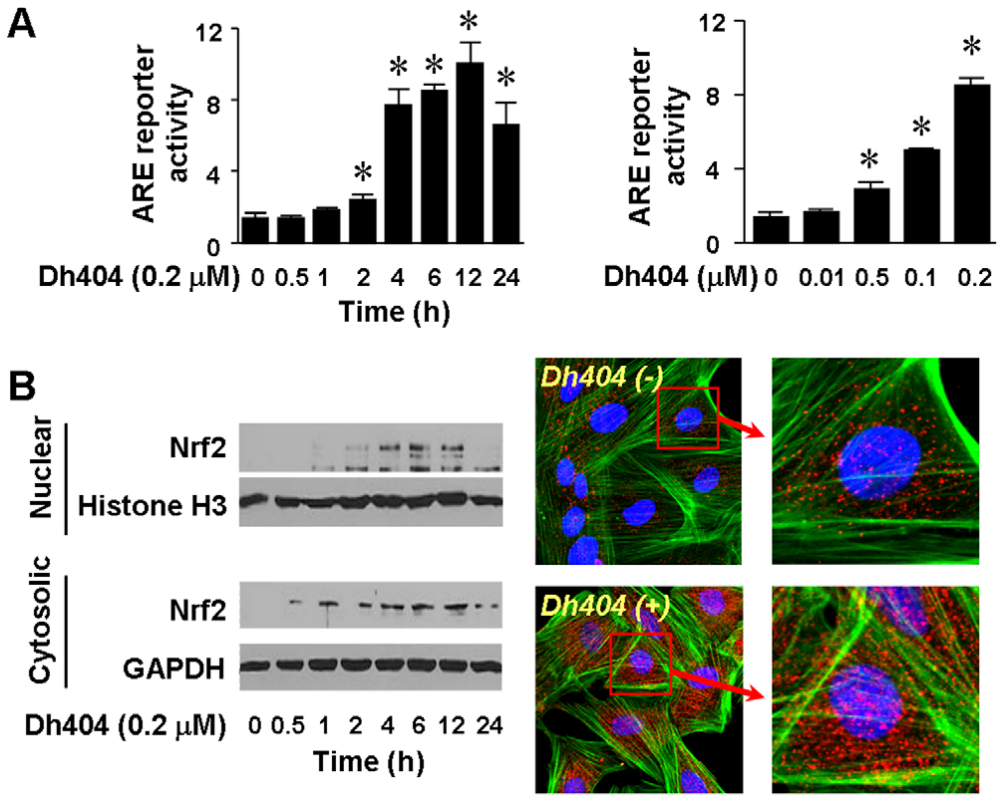
Dh404-induced activation of Nrf2 in H9C2 cardiomyocyte. Sub-confluent cells were transfected with ARE-luc (200 ng) and pRL-TK (10 ng) for 24 hours, and then cultured with serum-free DMEM for another 24 hours. The quiescent cells were stimulated with dh404 as indicated, and then subjected to ARE reporter assays (*A*) as well as immunoblot analysis of cytosolic and nuclear Nrf2 expression (*B*, left panel) or immunochemical staining of Nrf2 (*B*, right panel).

To further explore the biological relevance of dh404-induced activation of Nrf2 signaling, we determined whether dh404 activates Nrf2 in H9C2 cardiomyocytes. As expected, dh404 stabilized Nrf2 protein, enhanced Nrf2 nuclear translocation, leading to an increase in Nrf2-driven transcriptional activity (Figure 6).

### Dh404 Inhibits Hypertrophic Agonist-Induced Formation of ROS and RNS in H9C2 Cardiomyocytes via the Activating of Nrf2

**Figure 7:**
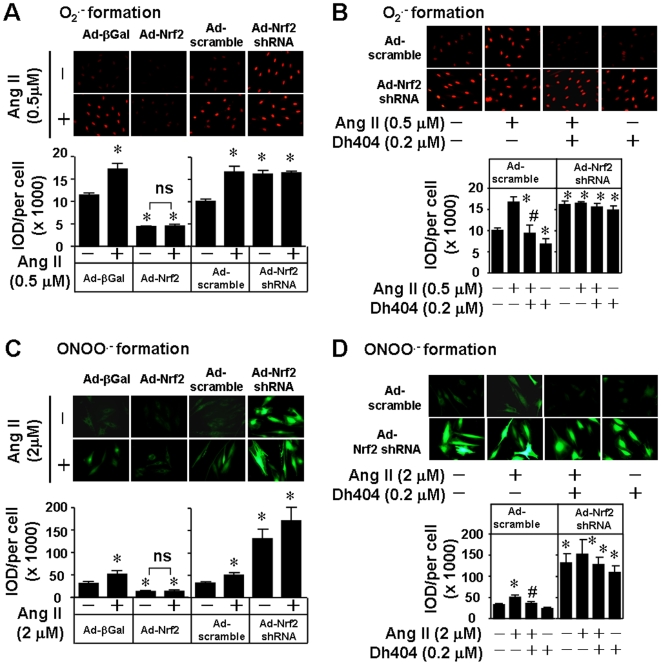
Dh404-induced inhibition of O_2_^.−^ and ONOO^−^ formation in H9C2 cardiomyocytes. *A*, Cells infected with 20 MOI of Ad-βGal, Ad-Nrf2, Ad-scramble or Ad-Nrf2 shRNA for 48 hours were stimulated with angiotensin II (Ang II, 0.5 µM) for 1 hour. *B*, Cells infected with 20 MOI of Ad-scramble or Ad-Nrf2 shRNA in the absence or presence of dh404 (200 nM) for 48 hours were stimulated with Ang II (0.5 µM) for 1 hour. *C*, Cells infected with 20 MOI of Ad-βGal, Ad-Nrf2, Ad-scramble or Ad-Nrf2 shRNA for 48 hours were stimulated with Ang II (2 µM) for 1 hour. *D*, Cells infected with 20 MOI of Ad-scramble or Ad-Nrf2 shRNA in the absence or presence of dh404 (200 nM) for 48 hours were stimulated with Ang II (2 µM) for 1 hour. O_2_^.−^ and ONOO^−^ formation was quantified as described in “*Materials and Methods*”. *p<0.05 vs control (−); #p<0.05 vs Ad-scramble Ang II (n = 8).

To determine a functional significance of the dh404-operated activation of Nrf2 signaling in cardiovascular system, we examined the effect of dh404 on oxidative stress in cardiomyocytes. It has been demonstrated in H9C2 cardiomyocytes that Ang II induces apoptosis via NADPH oxidase activation [36]. We observed that Ang II stimulation for 1 hour resulted in a dramatic increase in the formation of O_2_•^−^ and ONOO•^−^ in a dose-dependent manner with a peak of O_2_•^−^ and ONOO•^−^ production at concentration of 0.5 µM and 2 µM of Ang II respectively. However, Ang II did not stimulate H_2_O_2_ formation within a detectable range (data not shown). Adenoviral over-expression of Nrf2 inhibited both basal and Ang II-induced O_2_•^−^ and ONOO•^−^ formation in H9C2 cells, whereas adenoviral over-expression of Nrf2 shRNA exerted opposite effect (Figures 7A and C). The efficacy of adenoviral over-expression of Nrf2 and Nrf2 shRNA has been established in H9C2 cells (Figure S1). Of note, the loss of Nrf2 caused a robust increase in the formation of ONOO•^−^, a key player in the oxidative stress-mediated pathogenesis of various diseases including cardiovascular disorders [37], suggesting a crucial role of Nrf2 in cardiovascular protection. Importantly, dh404 was not only able to suppress the basal levels but also block the Ang II-induced formation of O_2_•^−^ and ONOO•^−^ in H9C2 cells. However, the inhibitory effects of dh404 on oxidative stress were essentially absent in the cells with Nrf2 knockdown (Figures 7B and D), demonstrating that Nrf2 is an essential mediator for dh404-induced suppression of oxidative stress.

### Dh404 Activates Nrf2 in the Heart

**Figure 8:**
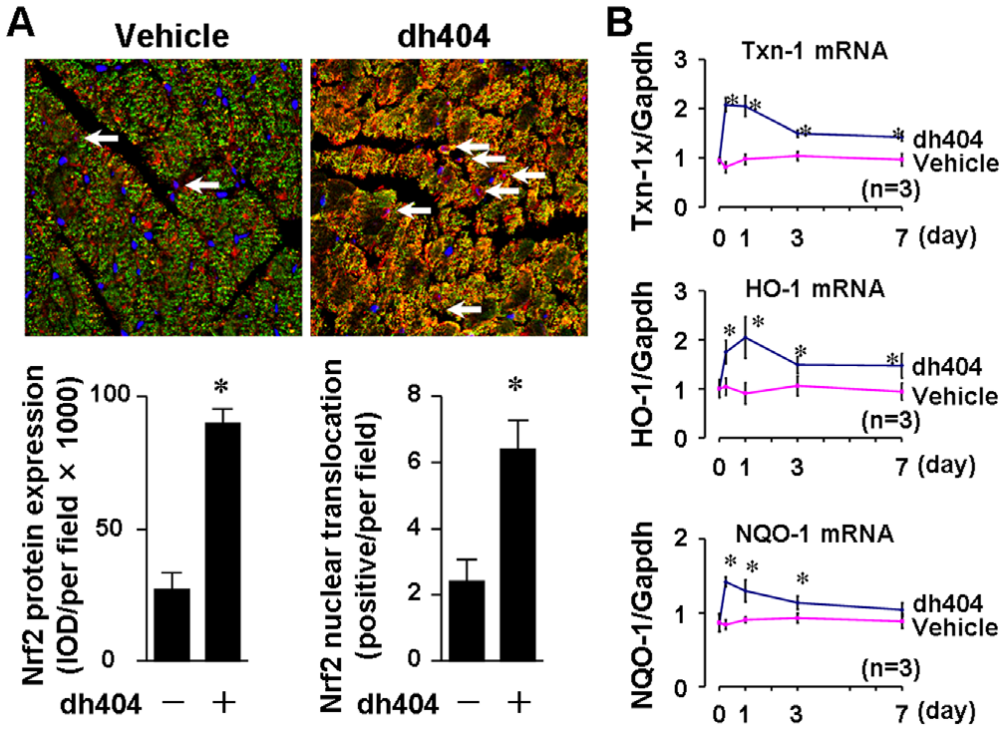
Dh404-mediated Nrf2 activation in rat neonatal cardiomyocytes and murine hearts. *A*, Upper panel; left ventricular sections from mice (n = 3) treated with vehicle or dh404 (50 mg/kg) for 6 hours. Nrf2 expression and nuclear translocation were analyzed using confocal microscopy. Red is Nrf2; Green is cardiac myosin heavy chain; Blue is nuclei. Lower panel; semi-quantification of Nrf2 expression was performed by measuring integrated optical density (IOD)s of red signal of Nrf2 staining in 4 randomly chosen fields in each tissue section. Two sections from each mouse treated with or without dh404 were randomly chosen for analysis. *B*, Q-PCR analysis of dh404-induced expression of Nrf2 target genes in the heart. *p<0.05 vs vehicle, n = 3.

Finally, we explored whether dh404 activates Nrf2 in a primary culture of cardiac myocytes and in the heart. As shown in Figure S2, dh404 treatment led to a robust up-regulation of Nrf2 in rat neonatal cardiomyocytes. While basal nuclear expression of Nrf2 is hardly detected, a dramatic increase in nuclear expression of Nrf2 was observed in dh404-treated cells. A single oral administration of dh404 significantly enhanced myocardial expression of Nrf2 with the majority localized in cardiac myocytes (Figure 8A). Moreover, oral dh404 administration activated several Nrf2 downstream genes in the heart (Figure 8B), including heme oxygenase-1 (HO-1), NAD(P)H:quinone oxidoreductase (NQO)-1, and thioredoxin-1 (Txn-1) that are important for Nrf2-mediated cardiac protection [24]. These results clearly demonstrate that dh404 activates Nrf2 in the heart, suggesting that dh404 might exert cardioprotective effects via activating Nrf2 signaling.

## Discussion

A therapeutic potential of natural triterpenoid derivatives for cardiovascular disease has been realized for thousands of years [1], [3], [38]. However, pharmaceutical approaches utilizing triterpenoid derivatives for the treatment of cardiovascular diseases have yet to be established because of the complex nature of mechanisms underlying their broad biological actions. In the present study, we report that a novel synthetic triterpenoid derivative, dihydro-CDDO-trifluoroethyl amide (2-cyano-3,12-dioxooleana-1-ene-28-trifluoroethylamide) or dh404, interacts with Cys-151 of Keap1 and leads to the suppression of the Keap1/Cul3/Rbx1 complex-mediated ubiquitination and degradation of Nrf2 proteins to enhance Nrf2 nuclear translocation and subsequent transcriptional events. Notably, dh404 suppresses oxidative stress via activating Nrf2 in cardiomyocytes. A single oral administration of dh404 was found to dramatically enhance Nrf2 activity, leading to a sustained activation of the cardioprotective genes that are driven by Nrf2 [24]. Therefore, our results demonstrate that dh404 represents a unique class of Nrf2 activators with a therapeutic potential for the treatment of cardiovascular diseases.

With regards to the molecular mechanism by which dh404 activates Nrf2, several intriguing issues remain to be addressed. Firstly, the dh404-induced Nrf2 nuclear accumulation may involve other regulator mechanisms, such as nuclear dissociation of Nrf2 with Keap1or nuclear turnover of Nrf2 [35], [39]. Secondly, dh404 might activate kinases to phosphorylate and stabilize Nrf2 as reported for other chemical inducers [10]–[12]. Finally, other Cys residues such as Cys-275 of Keap1 [25] might contribute to the dh404-mediated activation of Nrf2.

It is noteworthy that dh404 is a unique Nrf2 activator. Unlike dh404, we observed that oltipraz, another established Nrf2 activator, induced dramatic increases in superoxide and peroxynitrite generation in cardiomyocytes (Figure S3). Surprisingly, the levels of these free radicals in cardiomyocytes treated with oltipraz were even higher than that treated with angiotensin II, a most important factor in the pathogenesis of hypertrophic cardiomyopathy [40], [41]. Although it is unknown whether or not the strong side effects observed in clinical trials of oltipraz in cancer chemoprevention [42] is caused by the unexpected pro-oxidative effect of oltipraz, clearly the ability of oltipraz to treat cardiovascular diseases seems to be limited. In contrast, CDDO-Me, a drug that activates Nrf2-driven signaling against oxidative stress and inflammatory responses [43] is in late-stage clinical trails for the treatment of chronic kidney diseases. Thus, further characterization of dh404-mediated activation of Nrf2 signaling in the heart will thus facilitate the development of novel therapeutic strategies for the treatment of cardiac diseases.

**Figure 9:**
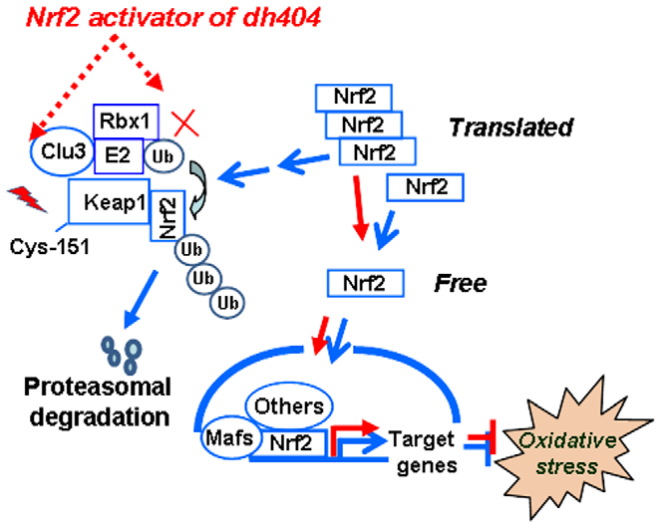
A model of dh404-induced activation of Nrf2. Under normal conditions, a single Keap1 protein is able to target multiple Nrf2 proteins for destruction via ubiquitin-proteasome system, and only a small portion of Nrf2 proteins is translocated into the nuclus to activate Nrf2-driven transcription. Dh404 interacts with Cys-151 of Keap1 to inhibit the Keap1-dependent degradation of Nrf2. Thus, newly synthesized Nrf2 proteins saturate the capacity of Keap1 binding with Nrf2, accumulate in the cytoplasm and subsequently translocate into the nucleus, thereby facilitating Nrf2-driven transcription of antioxidant genes to protect against oxidative stress.

In summary (Figure 9), we propose that, under normal conditions, a single Keap1 protein is able to target multiple Nrf2 proteins for destruction via the ubiquitin-proteasome system. However, when the ability of Keap1 to efficiently target Nrf2 proteins for degradation is disturbed, the capacity of Keap1 binding to Nrf2 is dramatically reduced [27]. Dh404 reacts with Cys-151 of Keap1 to inhibit the Keap1-dependent Nrf2 degradation. Thus, newly synthesized Nrf2 proteins will saturate the binding capacity of Keap1, and results in additional free Nrf2 proteins that accumulate in the cytoplasm, thereby triggering Nrf2 nuclear translocation and subsequent transcription, such as expression of antioxidant genes to suppress oxidative stress. Our results also demonstrate that dh404 suppresses oxidative stress via the activating of Nrf2 signaling in cardiomyocytes, as well as support the notion that dh404 as a novel Nrf2 activator possesses potential for the treatment of heart disease.

## Materials and Methods

### Animals and Treatment

#### Ethics statement

All procedures involving rat and mice were conducted in accordance with National Institutes of Health regulations and guidelines for the use and care of experimental animals. All animalprotocols were approved by the University of South Carolina Institutional Animal Use and Care Committee.

Male ICR mice were purchased from Jackson laboratory and housed under standard conditions in the Institution's AAALAC approved animal facility. Mice at 8 weeks of age were treated with a single gavage administration of vehicle or dh404 (50 mg/kg). Hearts were subsequently harvested at different time. Left ventricular section sections and RNAs were prepared for immunochemical staining and quantitative real time polymerase chain reaction (Q-PCR) analysis as previously described [24].

### Materials

A novel synthetic triterpenoid, dihydro-CDDO-trifluoroethyl amide (dh404) of 2-cyano-3,12-dioxooleana-1-ene-28-trifluoroethylamide, was synthesized, purified, and analytically characterized using similar methods as previously described [44]. The chemical structure is illustrated in Figure 1. Plasmids expressing wild-type Keap1, the chitin binding domain of the *Bacillus circulans* chitinase A1 gene (CBD)- tagged wild-type Keap1 (CBD-Keap1), Keap1 mutants of cysteine-to-serine mutations (C151S, C273S, and C288S), wild-type Nrf2, hemagglutinin (HA)-tagged wild-type Nrf2 (HA-Nrf2), HA-tagged ubiquitin (HA-Ub), HA-Cul3, Myc-Rbx1, or ARE firefly luciferase reporter (pARE-Luc) were prepared as previously described [26], [27]. The *Renilla* luciferase expression plasmid, pRL-TK, was purchased from Promega. Adenovirus of murine Nrf2 and rat Nrf2 shRNA were purchased from Wegen Inc. Protein G-Sepharose 4B, dimethyl sulfoxide (Me_2_SO), cycloheximide (CHX), MG132, and angiotensin II (Ang II) were purchased from Sigma-Aldrich. Probes of 2′,7′-dichlorofluorescin diacetate (DCFH-DA), dihydroethidium (DHE) and dihydrorhodamine-123 (DHR-123) were purchased from Molecular Probe (Eugene, OR).

### Cell Culture, Adenoviral Infection, Transfection and Reporter Gene Assay

NIH 3T3, HEK293, and H9C2 cells purchased from the American Type Culture Collection were cultured in Dulbecco's modified Eagle's medium (DMEM) supplemented with 10% fetal bovine serum (FBS). Rat neonatal cardiac myocytes were isolated and cultured in high glucose DMEM supplemented with 8% horse serum, 5% new-born calf serum as previously described [24]. H9C2 cells at ∼80% confluent status were infected with 20 MOI of adenovirus of beta-galactosidase (Ad-βGal), Nrf2 (Ad-Nrf2), control scramble shRNA (Ad-scramble) and rat Nrf2 shRNA (Ad-Nrf2 shRNA) for 48 hours in serum-free DMEM. Transfections were performed with Lipofectamone 2000 (Invitrogen) according to the manufacture's instruction. Each transfection was performed in triplicate at least three times. Reporter assay was determined using a dual luciferase assay kit (Promega) as previously described [45].

### Immunofluorescence Assays

NIH 3T3 cells were grown on glass coverslips, transfected with the HA-Nrf2 and the CBD-Keap1 expression plasmids with a ratio of 10∶1 and then fixed with 4% paraformaldehyde at 4°C overnight. Immunofluorescence staining was performed with rabbit anti-CBD antibodies (S6654S, New England Biolabs Inc.) and mouse anti-HA antibodies (F-7, sc-7392, Santa Cruz Biotechnology Inc.). H9C2 cells infected with Ad-Nrf2 or Ad-Nrf2 shRNA and rat neonatal cardiomyocytes treated with vehicle or dh404 (0.2 µM) for 1 hours were fixed as mentioned above, and stained with primary antibodies of Nrf2 (C-20, sc-722, Santa Cruz Biotechnology Inc.). F-actin was stained by Alexa Fluor® 488 phalloidin (Invitrogen). Cardiac myosin heavy chain was stained using mouse monoclonal anti-cardiac myosin heavy chain (ab15, Abcam Inc.). A standard protocol of immunochemical staining was described in Supplemental Methods S1. Confocal microscopic analysis was performed as previously described [24].

### Immunoprecipitation and Immunoblot Analysis

Cell lysates were subjected to immunoprecipitation and immunoblot analysis as previously described [46]. Nuclear and cytoplasmic subcellular fractions were obtained using NE-PER Nuclear and Cytoplasmic Extraction Reagents (Pierce) following the manufacturer's instructions. Primary antibodies of Nrf2 (H-300, sc-13032), HA (F-7 sc-7392), Keap1 (E-20 sc-15246), GAPDH (FL-335, sc-25778), ubiquitin (P4D1, sc-8017) and Histone H3 (FL-136, sc-10809) were purchased from Santa Cruz Biotechnology. An antibody of Myc (9b11, 2276) was purchased from Cell Signaling Technology. An antibody of CBD (S6654S) was purchased from New England Biolabs Inc.

### Measurement of Intracellular ROS and RNS

**G**eneration of ROS and RNS was assessed by the measurement of superoxide (O_2_•^−^), hydrogen peroxide (H_2_O_2_), and peroxynitrite (ONOO^−^) generation using the fluorescent probes DHE [47], DCFH-DA [48] and DHR-123 [49], respectively. DHE, a nonfluorescent membrane-permeant probe, interacts with O_2_•^−^, leading to the liberation of membrane-impermeant ethidium cations that fluoresce on intercalating with nuclear DNA. DCFH-DA diffuses through the cell membrane and is hydrolyzed by intracellular esterases to nonfluorescent dichlorofluorescin (DCFH) that is rapidly oxidized to highly fluorescent dichlorofluorescein by ROS such as hydrogen peroxide (H_2_O_2_), hydroxyl radical (·OH), and hydroperoxides (ROOH). DHR-123 is oxidized by ONOO^−^ to the highly fluorescent product rhodamine in vitro. For each experiment, 8 fields were randomly chosen to photograph, and integrated optical density (IOD)/per cell of the images was quantified with Image Pro Plus software. Triplicate experiments were repeated four times.

### Statistical Analysis

Values are expressed as mean ± S.E.M. The data were analyzed using analysis of variance with the Newman-Keuls' test unless specified. Values of *p*<0.05 were considered to be statistically significant.

## Supporting Information

Methods S1 (0.03 MB DOC)

Figure S1 Efficacy of adenoviral over-expression of Nrf2 and Nrf2 shRNA in H9C2 cardiomyocytes. A, Cells infected with different MOI of adenovirus of beta-galactosidase (Ad-βGal) and Ad-Nrf2 for 48 hours, and then subjected to Western blot analysis (Left panel) and immunochemical staining of Nrf2 (Right panel, 20 MOI for Ad-βGal or Ad-Nrf2). Western blot analysis demonstrated that Ad-βGal did not affect Nrf2 protein expression (data not shown). Red is Nrf2; Green is F-actin; Blue is nuclei. B, Cells infected with different MOI of Ad-scramble or Ad-Nrf2 shRNA 48 hours, and subjected to Q-PCR analysis of Nrf2 mRNA levels (Left panel) and Nrf2 protein levels (Right panel, 20 MOI of Ad-scramble or Ad-Nrf2) *p<0.05 vs control (0), (n = 4). Q-PCR analysis demonstrated that Ad-βGal did not affect Nrf2 mRNA expression (data not shown). Red is Nrf2; Green is F-actin; Blue is nuclei.
(1.51 MB TIF)

Figure S2 Dh404 up-regulates Nrf2 in rat neonatal cardiomyocytes. Rat neonatal cardiomyocytes were treated with or without dh404 (0.2 µM) for 1 hours, and then subjected to immunochemical staining as described in “Material and Methods”. Results are representative of three separated experiments. Red is Nrf2; Green is cardiac myosin heavy chain; Blue is nuclei.
(1.47 MB TIF)

Figure S3 Dh404-induced inhibition of O2.- and ONOO- formation in H9C2 cardiomyocytes. A, B, Cells were pretreated with oltipraz (20 µM) for 48 hours, and then were stimulated with Ang II at doses of 0.5 µM (O2.- measurement) or 2 µM (ONOO- measurement) for 1 hour. O2.- and ONOO- formation was quantified as described in “Material and Methods”. *p<0.05 vs control (-); #p<0.05 vs Ang II (+), (n = 8). ns, non-significant.
(0.31 MB TIF)
