# Supplementary material for: Dihydro-CDDO-Trifluoroethyl Amide (dh404), a Novel Nrf2 Activator, Suppresses Oxidative Stress in Cardiomyocytes
Source: PLoS One. 2009 Dec 21;4(12):e8391. doi: 10.1371/journal.pone.0008391 (PMC2791441; doi:10.1371/journal.pone.0008391)
Supplement: Methods S1 — (0.03 MB DOC) [file pone.0008391.s001.doc]

**SUPPLEMENTAL METHODS**

Nrf2 immunofluorescence was performed according to a standard protocol provided by Santa Cruz Biotechnology, Inc. Briefly, cells which were cultured in culture slides were fixed with 4% paraformaldehyde at 4ºC overnight, incubated in 0.1% Triton X-100 for 15 minutes, washed 3 times in PBS, then incubated in 5% normal goat serum for 30 minutes at room temperature. The slides were incubated with a rabbit anti mouse Nrf2 polyclonal antibody (C-20, sc-722, Santa Cruz Biotechnology, Inc.) at 4ºC overnight, with a biotinylated secondary antibody for 30 minutes at room temperature, with Streptavidin conjugated Alexa FluorR 594 (Cat# S11227, Sigma-Aldrich), and DAPI for 30 minutes at room temperature. F-actin was stained by Alexa Fluor® 488 phalloidin (Invitrogen). Cardiac myosin heavy chain was stained using mouse monoclonal anti-cardiac myosin heavy chain (ab15, Abcam Inc.). Images were acquired by confocal microscopy (LSM510META, Carl Zeiss Inc., Maple Grove, MN) at 630 × magnification.
