# Supplementary figures and images for: Dihydro-CDDO-Trifluoroethyl Amide (dh404), a Novel Nrf2 Activator, Suppresses Oxidative Stress in Cardiomyocytes
Source: PLoS One. 2009 Dec 21;4(12):e8391. doi: 10.1371/journal.pone.0008391 (PMC2791441; doi:10.1371/journal.pone.0008391)

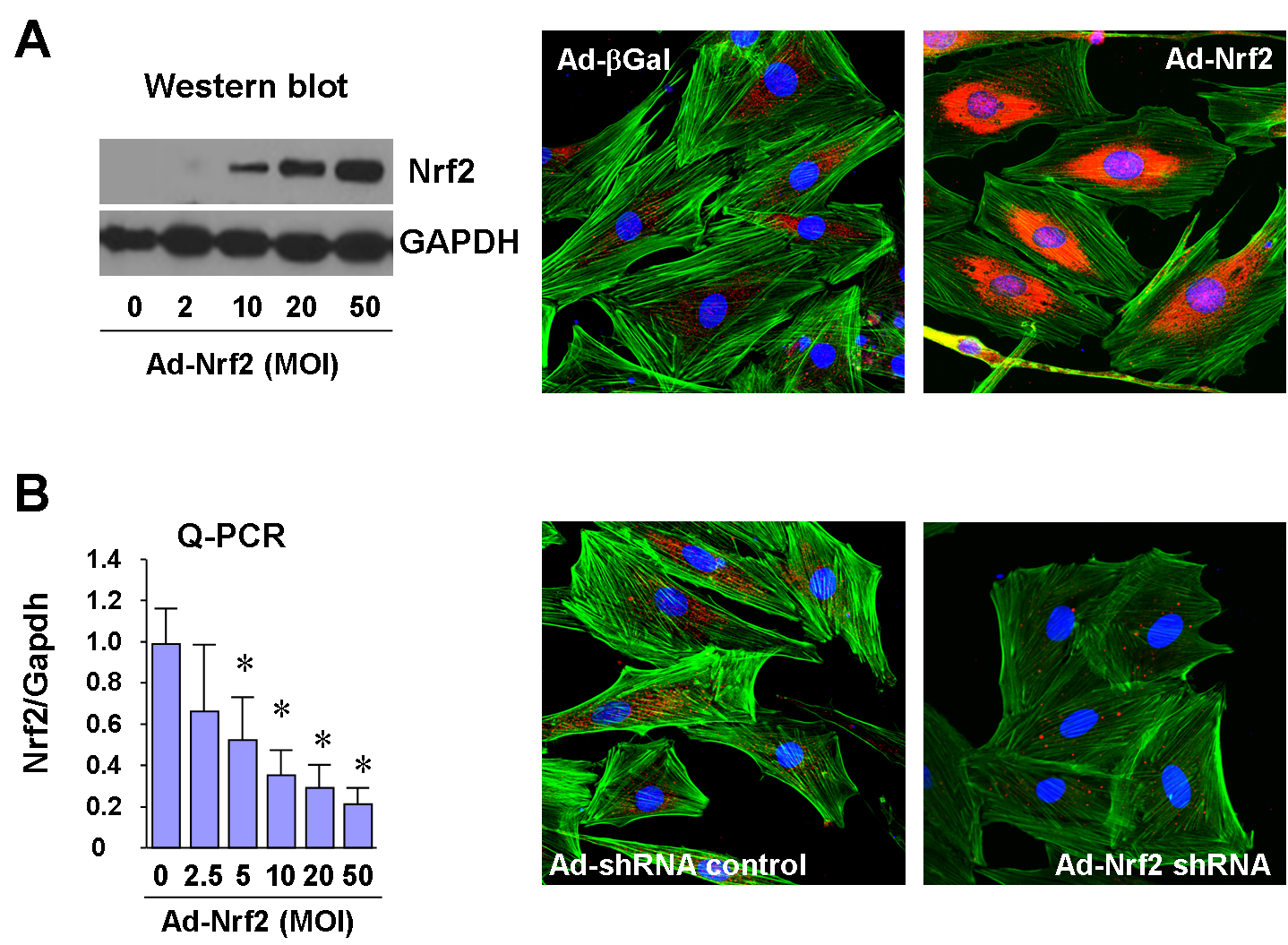

Supplement: Figure S1 — Efficacy of adenoviral over-expression of Nrf2 and Nrf2 shRNA in H9C2 cardiomyocytes. A, Cells infected with different MOI of adenovirus of beta-galactosidase (Ad-βGal) and Ad-Nrf2 for 48 hours, and then subjected to Western blot analysis (Left panel) and immunochemical staining of Nrf2 (Right panel, 20 MOI for Ad-βGal or Ad-Nrf2). Western blot analysis demonstrated that Ad-βGal did not affect Nrf2 protein expression (data not shown). Red is Nrf2; Green is F-actin; Blue is nuclei. B, Cells infected with different MOI of Ad-scramble or Ad-Nrf2 shRNA 48 hours, and subjected to Q-PCR analysis of Nrf2 mRNA levels (Left panel) and Nrf2 protein levels (Right panel, 20 MOI of Ad-scramble or Ad-Nrf2) *p<0.05 vs control (0), (n = 4). Q-PCR analysis demonstrated that Ad-βGal did not affect Nrf2 mRNA expression (data not shown). Red is Nrf2; Green is F-actin; Blue is nuclei. (1.51 MB TIF) [file pone.0008391.s002.tif]

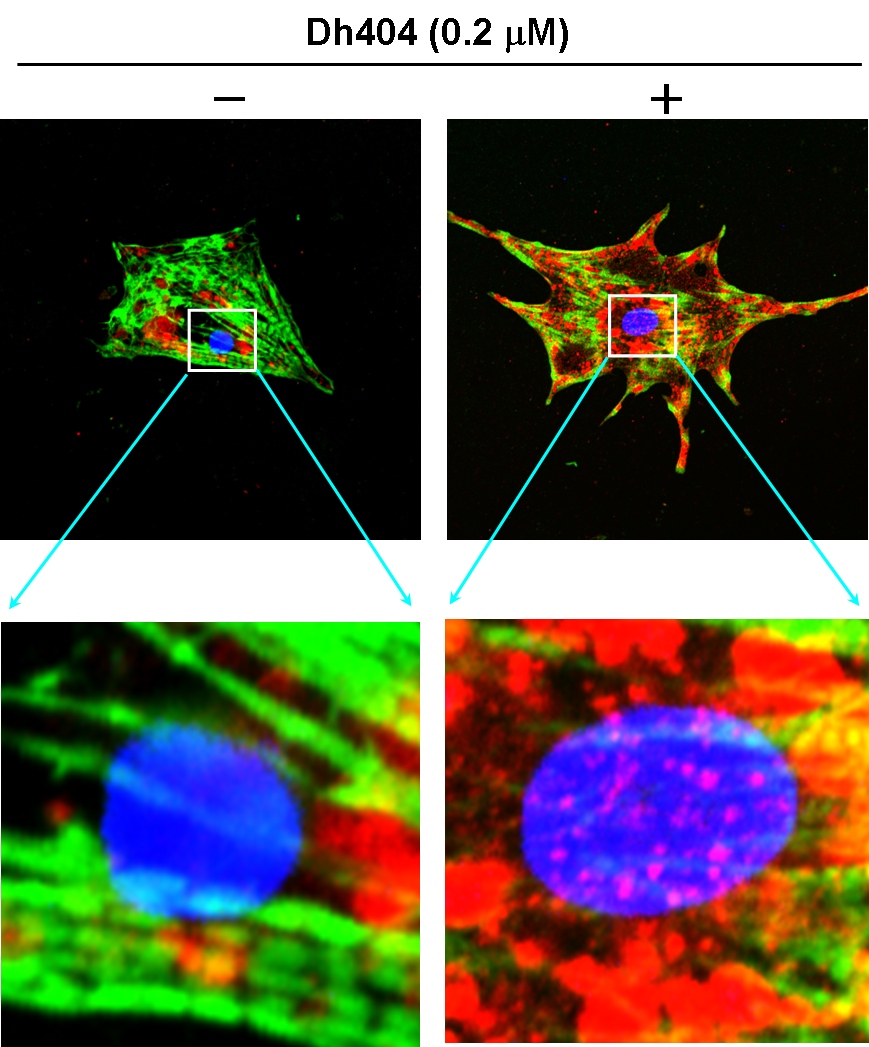

Supplement: Figure S2 — Dh404 up-regulates Nrf2 in rat neonatal cardiomyocytes. Rat neonatal cardiomyocytes were treated with or without dh404 (0.2 µM) for 1 hours, and then subjected to immunochemical staining as described in “Material and Methods”. Results are representative of three separated experiments. Red is Nrf2; Green is cardiac myosin heavy chain; Blue is nuclei. (1.47 MB TIF) [file pone.0008391.s003.tif]

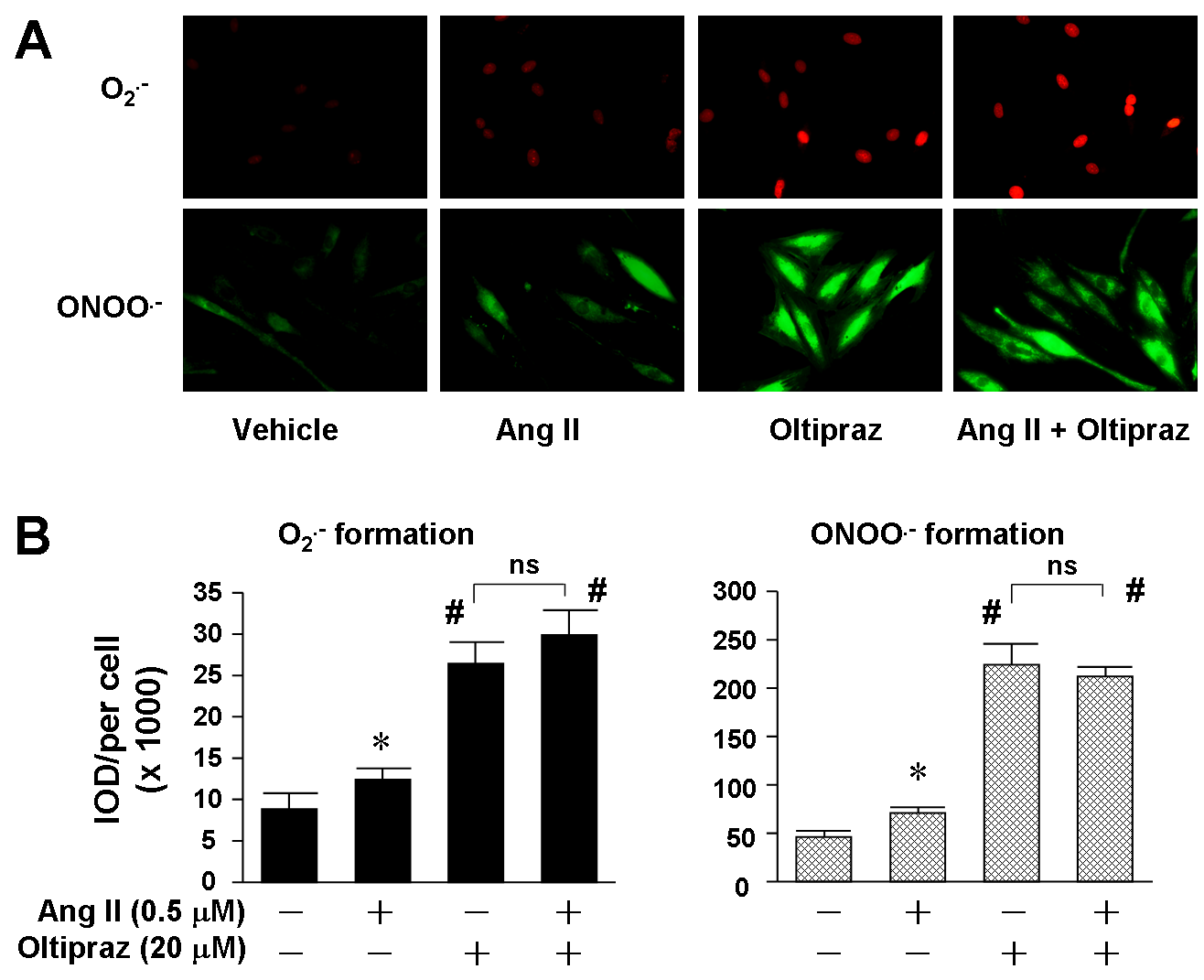

Supplement: Figure S3 — Dh404-induced inhibition of O2.- and ONOO- formation in H9C2 cardiomyocytes. A, B, Cells were pretreated with oltipraz (20 µM) for 48 hours, and then were stimulated with Ang II at doses of 0.5 µM (O2.- measurement) or 2 µM (ONOO- measurement) for 1 hour. O2.- and ONOO- formation was quantified as described in “Material and Methods”. *p<0.05 vs control (-); #p<0.05 vs Ang II (+), (n = 8). ns, non-significant. (0.31 MB TIF) [file pone.0008391.s004.tif]
